# Reconsolidation/destabilization, extinction and forgetting of fear memory as therapeutic targets for PTSD

**DOI:** 10.1007/s00213-018-5086-2

**Published:** 2018-10-29

**Authors:** Satoshi Kida

**Affiliations:** 1 Department of Bioscience, Faculty of Life Science, Tokyo University of Agriculture, Tokyo, Japan; 2 Department of Bioscience, Faculty of Applied Bioscience, Tokyo University of Agriculture, 1-1-1 Sakuragaoka, Setagaya-ku, Tokyo, 156-8502 Japan

**Keywords:** Extinction, Reconsolidation, Destabilization, Consolidation, Forgetting, PTSD, Fear memory

## Abstract

Post-traumatic stress disorder (PTSD) is a psychiatric disorder associated with memories of traumatic experiences. Conditioned fear memory, a representative model of traumatic memories, is observed across species from lower to higher animals, including humans. Numerous studies have investigated the mechanisms of conditioned fear memory and have led to the identification of the underlying processes involved in fear memory regulation, including cellular and systems consolidation of fear conditioning, destabilization/reconsolidation and extinction after fear memory retrieval, and forgetting of fear memory. These studies suggested that mechanisms for fear memory regulation are shared by humans and other higher animals. Additionally, rodent studies have identified the mechanisms of fear memory at the molecular, cellular, and circuit levels. Findings from these studies in rodents have been applied to facilitate the development and improvement of PTSD intervention. For instance, reconsolidation and extinction of fear memories have been applied for PTSD treatment to improve prolonged exposure (PE) therapy, an effective psychotherapy for PTSD. Combination of medications weakening retrieved traumatic memory (e.g., by facilitating both destabilization and extinction) with PE therapy may contribute to improvement of PTSD. Interestingly, a recent study in mice identified forgetting of fear memory as another potential therapeutic target for PTSD. A better understanding of the mechanisms involved in fear memory processes is likely to facilitate the development of better treatments for PTSD. This review describes fear memory processes and their mechanisms and discusses the pros and cons of applying how this knowledge can be applied in the development of interventions for PTSD.

## Fear memory and consolidation

Post-traumatic stress disorder (PTSD) is a mental disorder associated with traumatic memory, including fear memory. In experimental animals, Pavlovian fear conditioning has been widely used as a model of PTSD. The most widely used fear conditioning paradigms are based on a contextual or cued fear conditioning task, in which a rodent learns the association (i.e., training) between the conditioned stimulus (CS), such as a chamber (context) or a tone (cue), and an unconditioned stimulus (US) inducing fear, such as mild electrical foot shocks (Phillips and LeDoux 1992). Fear conditioning generates fear memory, reflecting CS-US association. Importantly, conditioned fear memory has been observed in many animal species from insects to humans (Knight et al. 2004; Lissek et al. 2008).

When re-exposed to the chamber (context) or the tone (cue), the conditioned rodent shows immobile freezing (fear) responses by retrieving the conditioned fear memory. Generally, fear memory is assessed based on the duration of the freezing response during re-exposure to the CS (chamber or tone) for a certain period of time (e.g., 3–5 min).

**Fig. 1:**
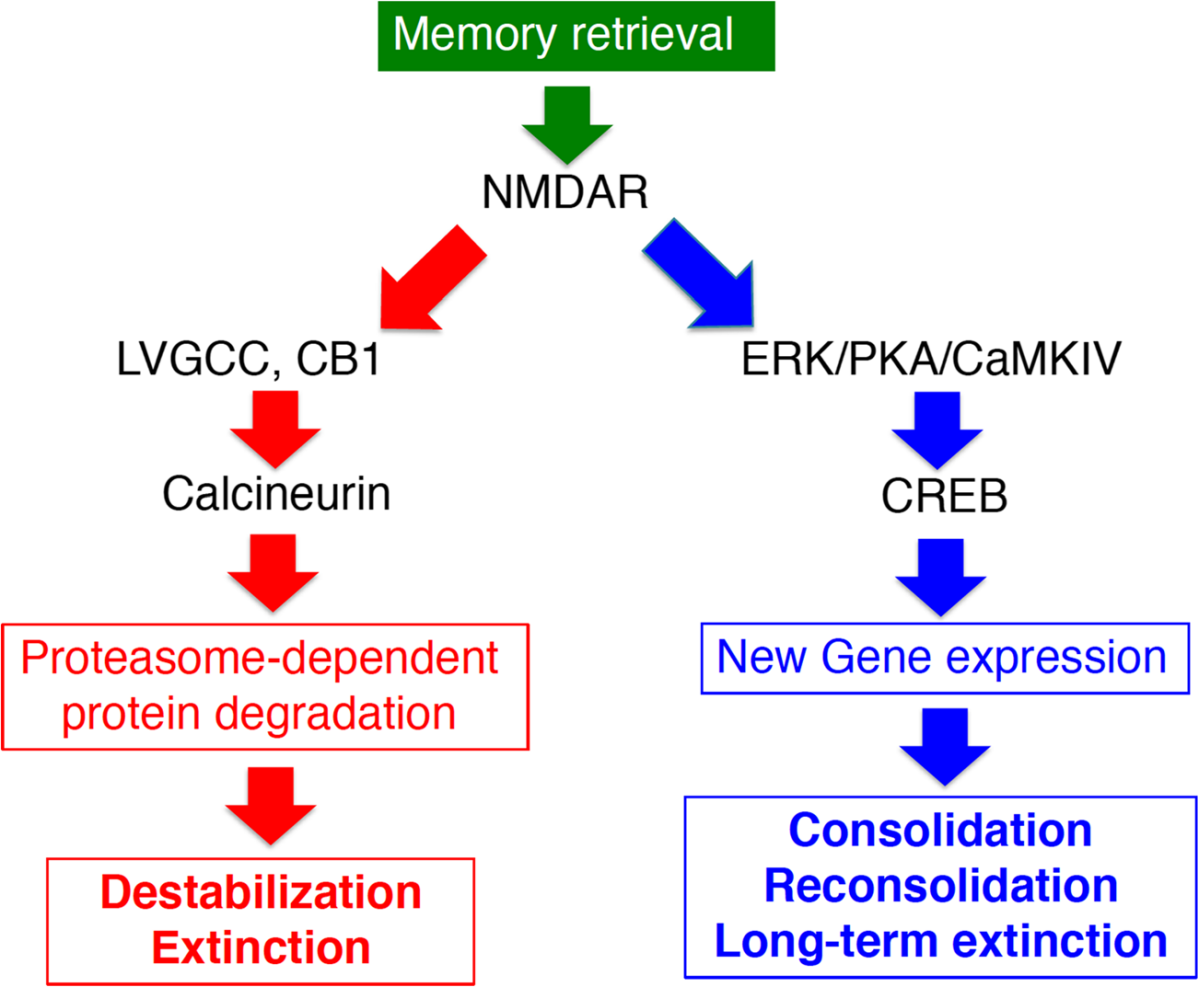
Signal transduction pathways for fear memory regulation. Memory destabilization or extinction after retrieval requires the activation of NMDA receptors (NMDAR) as the starting point; subsequent activation of L-type voltage-gated calcium channels (LVGCC), endogenous cannabinoid receptors (CB1), calcineurin, and finally proteasome-dependent protein degradation occurs. On the other hand, memory consolidation, reconsolidation, and consolidation of extinction memory (long-term extinction) require activation of transcription factor CREB-mediated transcription through phosphorylation at serine 133 by the activation of extracellular signal-regulated kinase/MAP kinase (ERK), protein A kinase (PKA), and/or Ca^2+^/calmodulin-dependent protein kinase IV (CaMKIV), which are downstream factors from cAMP and Ca^2+^ signal transduction

Long-term memory (LTM) is stable while short-term memory (STM; ~several hours) is labile. The process of stabilizing a labile STM is known as “memory consolidation” (Silva et al. 1998). Memory consolidation consists of two sequential but dissociable processes. The first is “cellular consolidation”, which is complete within a few hours to a day. During this process, the most critical biochemical feature of memory consolidation is the requirement for new gene expression leading to plastic and/or structural changes in the neural circuits including neurons and synapses, thereby enabling memory storage (Abel and Lattal 2001; Davis and Squire 1984; Flexner et al. 1965; McGaugh 2000; Silva et al. 1998). This new gene expression is activated by transcriptional regulation factor cAMP responsive element binding protein (CREB) and its upstream signal transduction factors such as protein kinase A (PKA) and Ca^2+^/calmodulin-dependent protein kinase IV, which regulate the activation of CREB (Kida et al. 2002; Kida and Serita 2014; Silva et al. 1998) (Fig.1). The second step is “systems consolidation”, which takes much longer (3 to 4 weeks in rodents) than cellular consolidation (Anagnostaras et al. 1999; Frankland and Bontempi 2005; Kim and Fanselow 1992). In systems consolidation, the degree of hippocampal dependency of memory retrieval decreases as time passes from initial memory formation, and remote memories can ultimately be retrieved even when hippocampal function is inhibited or disrupted. In other words, memories become more independent of the hippocampus and more dependent on the cerebral cortices. Interestingly, it is important to note that recent studies have raised the possibility that even remote memory remains hippocampus-dependent (Goshen et al. 2011; Wiltgen and Tanaka 2013).

## Destabilization and reconsolidation of fear memory

When a memory is retrieved, it returns to a labile state similar to that existing immediately after memory formation (destabilization) (Nader et al. 2000a, 2000b). Re-stabilization is necessary for the memory to be re-stored. This re-stabilization process requires the activation of gene expression as cellular consolidation does this; therefore, the process of re-storing memories is referred to as reconsolidation (Nader et al. 2000a, 2000b). Reconsolidation requires CREB-mediated gene expression and is regulated by molecular mechanisms similar to but at least partially distinct from those involved in cellular consolidation (Kida et al. 2002; Kida and Serita 2014; Lee et al. 2004; Tronson and Taylor 2007; Silva et al. 1998; von Hertzen and Giese 2005). Reconsolidation is thought to function in the updating of memories (Fukushima et al. 2014; Nader et al. 2000b; Tronel et al. 2005).

Inhibition of gene expression during memory reconsolidation leads to disruption of memory, suggesting that retrieved memories should have been in a state similar to that of short-term memories (i.e., labile) (Nader et al. 2000a, 2000b). Therefore, the molecular mechanism responsible for inducing destabilization of retrieved memory has been examined by preventing memory disruption through the inhibition of new gene expression; memory disruption via inhibition of new gene expression would not occur if destabilization after retrieval was inhibited (i.e., memories that are not destabilized cannot be disrupted by the inhibition of gene expression). To date, GluN2B NMDA receptor, proteasome-dependent protein degradation, L-type voltage-gated calcium channels, Ca2+/calmodulin-dependent protein kinase II (CaMKII), cannabinoid receptors CB1, calcineurin, muscarinic receptors, and dopamine D1/D5 receptors have been identified as being necessary for memory destabilization after retrieval (Ben Mamou et al. 2006; Fukushima et al. 2014; Jarome et al. 2016; Kim et al. 2011; Lee et al. 2008; Merlo et al. 2015; Milton et al. 2013; Stiver et al. 2015; Suzuki et al. 2008; Vigil et al. 2017) (Fig.1).

Furthermore, it has been suggested that endocytosis of GluA2-containing calcium-impermeable-AMPA receptors at lateral amygdala post-synaptic membranes is required for destabilization of cued fear memory after retrieval, while activation of calcium-permeable AMPA receptors that do not contain GluA2 is necessary for reconsolidation of the memory (Hong et al. 2013). Additionally, it has been shown that memory retrieval does not always lead to destabilization/reconsolidation and that whether or not destabilization occurs depends on the strength and age of the memory as well as the duration of memory retrieval (Suzuki et al. 2004). For instance, it has been shown that remote/old contextual fear memory undergoing systems consolidation and strong fear memory generated by strong or repeated electric shocks are resistant to memory destabilization, since normal re-exposure procedure (e.g., 3 min re-exposure for contextual fear memory) is insufficient for the induction of destabilization but long-term re-exposure (e.g., 10 min) is required to induce it (reconsolidation boundaries). These findings demonstrate that destabilization is an active memory process underlying the molecular mechanisms regulated by extracellular and intracellular signal transduction pathways. Induction of the destabilization process may open a window to modify and update an original memory after retrieval.

## Fear memory extinction

Fear response such as freezing in fear conditioning elicited by fear memory retrieval is a conditioned reflex. Therefore, continuous re-exposure to the CS without the US induces fear responses (freezing) initially but gradually less so over time. This phenomenon is defined as “memory extinction” (Myers and Davis 2002). A previous study showed that in the contextual fear conditioning task, a long re-exposure to the conditioned chamber (30 min) induces learning for extinction (Suzuki et al. 2004). Pavlov first pointed out the presence of memory extinction in 1927 (Pavlov 1927). It is important to note that fear memory extinction is not simply the elicitation of eliciting forgetting, erasure, or disruption of fear memory. Rather, it is a new inhibitory memory acquired by a learning process through re-exposure to the CS, as indicated by previous observations that fear memory is not erased when fear memory is extinguished. For instance, fear responses are recovered when animals are re-exposed to the CS after a long time (e.g., 1 month) following extinction learning (i.e., spontaneous recovery) (Myers and Davis 2002; Rescorla 2004; Schiller et al. 2008). Fear memory is thought to be extinguished by inhibitory circuits suppressing fear responses. Furthermore, extinction memories are consolidated (stored) by a molecular mechanism similar to consolidation such as requirement for new gene expression and activation of CREB-mediated transcription (Mamiya et al. 2009; Santini et al. 2004). Consolidation of contextual fear extinction requires gene expression in the mPFC and amygdala, whereas consolidation of contextual fear memory does it in the hippocampus and amygdala (Mamiya et al. 2009).

## PTSD treatment strategies targeting memory processes after retrieval

Findings from rodent studies have been applied to the development of PTSD treatment since the mechanisms for fear memory regulation may be similar between humans and other animals (Knight et al. 2004; Phillips and LeDoux 1992). Prolonged exposure (PE) therapy is known to be an effective cognitive therapy for PTSD (Bentz et al. 2010; Foa and Kozak 1986; Mueller and Cahill 2010). In PE therapy, PTSD is improved by repeatedly and continuously having the patient retrieve vividly the traumatic experience with a therapist/physician. The biological basis for PE therapy is thought to involve extinction of fear memory (Davis et al. 2006; Kaplan and Moore 2011; Mueller and Cahill 2010).

**Fig. 2:**
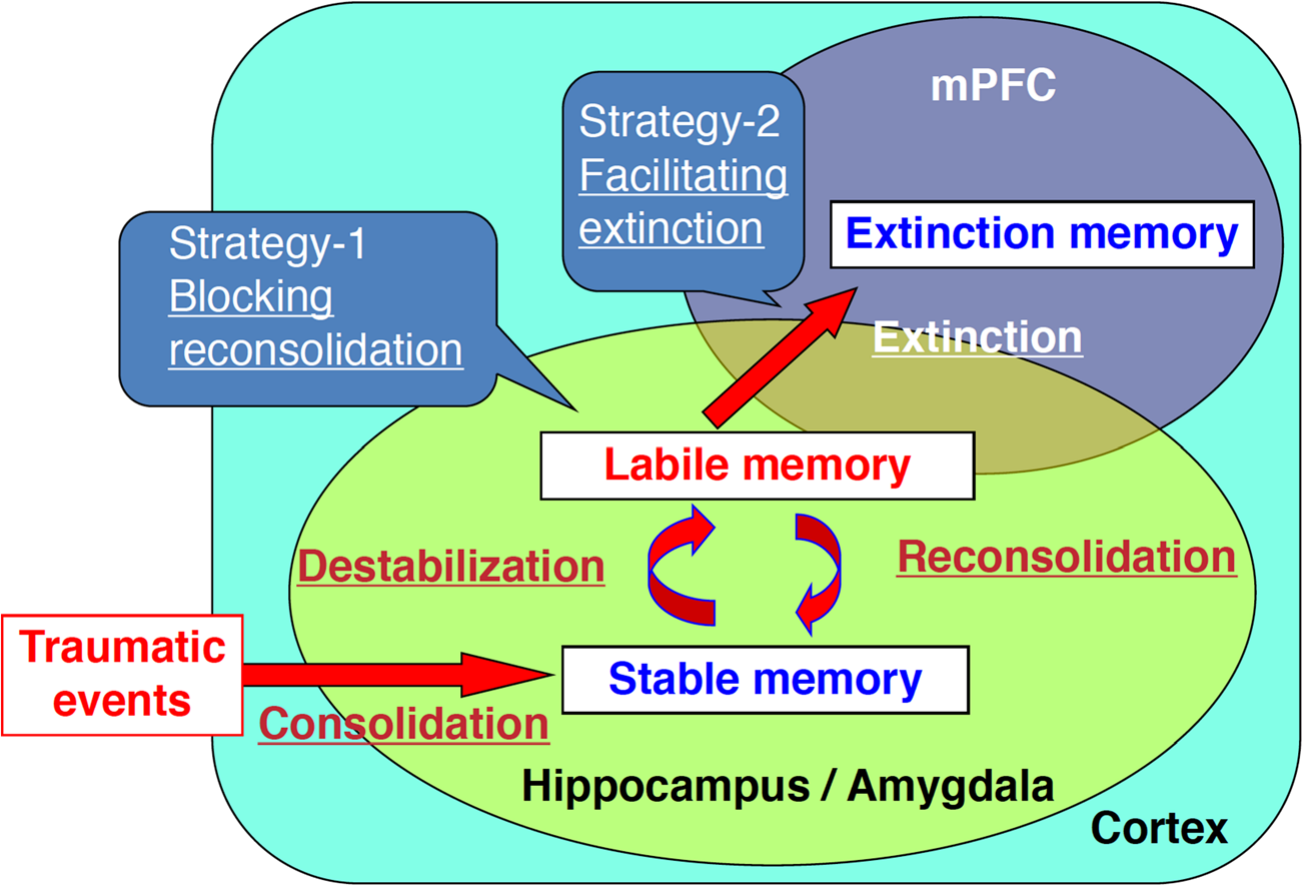
Fear memory regulation and PTSD treatment strategies. Novel PTSD therapy methods that shorten the duration of exposure therapy by blocking reconsolidation of fear memory, or by facilitating fear memory extinction, are being developed

A disadvantage of the PE method is that it is difficult to treat a large number of patients because the therapist/physician treats a patient through one-on-one sessions over a long period of time. Additionally, rates of drop-out as well as long-term relapse should be concerned during and following the PE therapy, respectively. Therefore, it is important to develop methods to shorten the duration of exposure therapy. To do this, methods have been proposed that artificially regulate reconsolidation/destabilization or extinction to weaken traumatic memory and phobia in combination with PE therapy (Brunet et al. 2008; Debiec and LeDoux 2006; Kaplan and Moore 2011; Litz et al. 2012; Rauch et al. 2006; Richardson et al. 2004; Soeter and Kindt 2015). Specifically, disruption of fear memory by blocking the reconsolidation or facilitation of fear extinction using medications has been attempted as a treatment for PTSD in combination with PE therapy (Fig. 2).

Remarkably, PTSD pathologies such as fear responses to traumatic events are reduced by the administration of the β-adrenergic blocker propranolol, perhaps, through targeting reconsolidation (Brunet et al. 2008). A recent study reported that administration of propranolol following the presentation of a tarantula ameliorated the severity of phobia in patients with arachnophobia (Soeter and Kindt 2015). In addition, D-cycloserine, a partial agonist of NMDA glutamate receptors, has been used to shorten the duration of PE therapy by enhancing memory extinction, since this drug has been shown to promote fear memory extinction in rodents. Clinical studies have suggested its efficacy (Davis et al. 2006; de Kleine et al. 2015; Mueller and Cahill 2010; Rauch et al. 2006; Richardson et al. 2004; but see Litz et al. 2012).

Solutions for spontaneous recovery of fear response observed even after extinguishing fear memory have also been considered. Interestingly, previous study using rodents has shown that fear responses did not return when extinction was induced within a time window when fear memories are in a state of reconsolidation/destabilization (reconsolidation phase) following (within a few hours) retrieval of the memory (reconsolidation-update) (Monfils et al. 2009; but see Luyten and Beckers 2017). Similar prevention of spontaneous recovery of fear response has been observed in humans (Schiller et al. 2010). Additionally, a recent study showed that spontaneous recovery was prevented by repeated extinction training, suggesting that spontaneous recovery of fear responses is due to insufficient extinction learning (An et al. 2017). This experimental evidence will help to develop or modify methods for PTSD treatment targeting fear memory extinction.

Although PTSD treatment targeting reconsolidation and extinction based on animal studies has been attempted, as described above, concerns about these approaches have arisen. For instance, inhibition of gene expression can be expected to disrupt retrieved fear memory in the reconsolidation phase when fear memory is destabilized following retrieval (Kida et al. 2002; Mamiya et al. 2009; Nader et al. 2000a). In contrast, fear memory remains intact in the extinction phase when fear memory is extinguished but gene expression is inhibited, since the inhibition of gene expression blocks consolidation of fear memory extinction (long-term extinction) but does not affect fear memory (Mamiya et al. 2009; Santini et al. 2004). Similarly, D-cycloserine can enhance retrieved fear memory in the reconsolidation phase while it facilitates extinction of fear memory in the extinction phase (Lee et al. 2006). Therefore, if drugs show opposite effects on reconsolidation and extinction, memory phases during PE therapy should be carefully estimated for during reconsolidation or extinction phase. Interestingly, recent studies have suggested a transition period from reconsolidation to extinction phases of fear memory (Cassini et al. 2017; Merlo et al. 2014). This finding may help to find a way to discriminate reconsolidation and extinction phases. Additionally, it is important to identify biological markers that can aid in the estimation of the reconsolidation and extinction memory phases.

Previous studies have investigated the molecular signatures of reconsolidation/destabilization and extinction. Importantly, these studies identified molecules required for both extinction and destabilization. Activation of calcineurin, L-type voltage-gated calcium channels, cannabinoid CB1 receptors, and proteasome-dependent protein degradation induce memory destabilization after retrieval, and activation of these factors is also required for fear memory extinction (Fukushima et al. 2014; Kim et al. 2011; Lee et al. 2008; Marsicano et al. 2002; Suzuki et al. 2004, 2008; but see Merlo et al. 2014). Thus, memory destabilization and extinction are induced, at least in part, through the activation of common molecules/signal transduction pathways. Activation of these molecules would therefore attenuate fear memory in the reconsolidation phase by promoting destabilization of fear memory, while it would facilitate extinction in the extinction phase. Drugs that activate these molecules are thought to be good candidates for shortening PE therapy since activation of these molecules would negatively regulate fear memory regardless of the memory phase of reconsolidation or extinction. It is important to identify additional target molecules that would enable the use of existing medications that activate both destabilization and extinction of fear memory.

## PTSD treatment strategy targeting fear memory forgetting

Herman Ebbinghaus characterized the forgetting curve in humans and argued that human memories would be forgotten with the passage of time (Ebbinghaus 1913). There is growing evidence to support that forgetting is an active memory process. Importantly, a recent finding showed that forgetting of hippocampus-dependent memory is facilitated through an increase in adult hippocampal neurogenesis (Akers et al. 2014). Hippocampal neurogenesis may contribute to “memory clearance” through remodeling that degrades memories already stored in the hippocampus (Frankland and Josselyn 2016). Therefore, this finding raises the possibility that the facilitation of forgetting by hippocampal neurogenesis is applicable to PTSD treatment although the controversial observations in persistence of human hippocampal neurogenesis were reported (Boldrini et al. 2018; Sorrells et al. 2018).

**Fig. 3:**
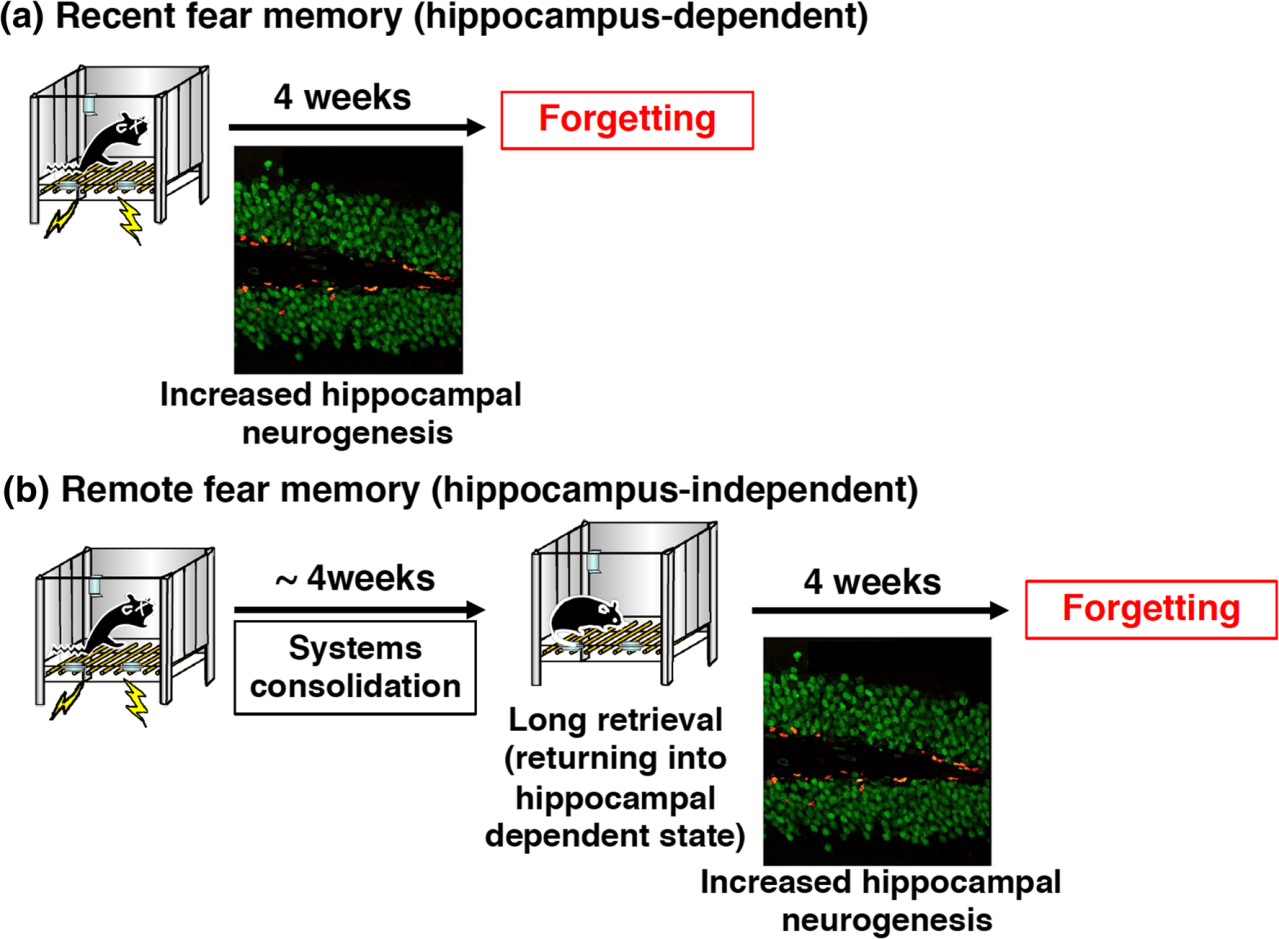
PTSD treatment strategies with fear memory forgetting as a target. **a** Forgetting of hippocampus-dependent fear memories is enhanced through increased hippocampal neurogenesis. **b** Remote memories that are hippocampus-independent could return into a hippocampus-dependent state through long-duration retrieval of fear memory by long re-exposure to the context for 10 min, and then forgetting of the remote memories can be promoted through hippocampal neurogenesis.

Memantine (MEM) is an antagonist of the NMDA glutamate receptor and is a therapeutic agent for Alzheimer’s disease (Bormann 1989; Namba et al. 2009). Interestingly, MEM has been shown to be a neurogenesis enhancer that dramatically increases adult hippocampal neurogenesis (Maekawa et al. 2009; Ishikawa et al. 2014). Recent studies have shown that forgetting of contextual fear memory is promoted via increased adult hippocampal neurogenesis when mice were treated with MEM once a week for 4 weeks following the formation of contextual fear memory (Akers et al. 2014; Ishikawa et al. 2016) (Fig. 3A). A positive correlation between facilitation of forgetting and adult hippocampal neurogenesis was observed, supporting the conclusion that forgetting is facilitated via hippocampal neurogenesis (Ishikawa et al. 2016). It is important to point out that unlike the induction of extinction or reconsolidation, intervention such as a fear memory retrieval session triggered by re-exposure to the CS is not required during the MEM administration period; rather, the mice simply receive MEM by systemic injection every week for 1 month. This is advantageous for PTSD therapy since facilitating forgetting through treatment with a hippocampal neurogenesis enhancer may result in improvement of PTSD using medications without PE therapy.

It is important to note that MEM might block memory retention through inhibition of NMDA receptor activation (Shimizu et al. 2000). However, forgetting of fear memory was observed even when mice performed physical exercise for 1 month using a running wheel in a cage to promote hippocampal neurogenesis (Ishikawa et al. 2016; van Praag et al. 1999). The observation that exercise induces forgetting of fear memory supports our conclusion that the effect of MEM on forgetting of fear memory is attributable to increased hippocampal neurogenesis but not blocking memory retention by inhibiting the NMDA glutamate receptor.

It is important to note that PTSD is associated with old traumatic events and, therefore, methods to promote forgetting of remote traumatic memory should be considered as a means to improve PTSD treatment. In contrast to its effect on recent memory, MEM treatment failed to enhance forgetting of remote contextual fear memory (Ishikawa et al. 2016). However, this observation is not surprising. Increases in neurogenesis should only impact memories that depend on the hippocampus since remote contextual memory is independent of the hippocampus (Ishikawa et al. 2016). Therefore, induction of forgetting via increased hippocampal neurogenesis is necessary to facilitate forgetting of remote fear memory by neurogenesis enhancers in the treatment of PTSD. A previous study showed that reminders (e.g., context re-exposure) can render memories hippocampus-dependent again and make them vulnerable to amnestic treatments that block reconsolidation (e.g., protein synthesis blockade) (Debiec et al. 2002; Suzuki et al. 2004). These phenomena of destabilization/reconsolidation boundaries (see above) raise the possibility that prolonged (10 min) context re-exposures (long reminders) can render even remote contextual fear memories labile and hippocampus-dependent. Therefore, based on this same logic, the effects of MEM administration and exercise on remote contextual fear memory were examined following a long-duration context reminder (i.e., for 10 min but not 3 min) that induces destabilization/reconsolidation of remote memory (Ishikawa et al. 2016). As with recent memory, forgetting of remote contextual fear memory was enhanced by MEM administration or exercise following long-duration context reminders (Fig. 3B). The following experimental evidence supports the hypothesis that remote contextual fear memory returns to a hippocampus-dependent state after long-duration memory retrieval by an extended reminder: (1) hippocampal gene expression is observed when remote contextual fear memory was retrieved following a long (10 min) but not a short (3 min) reminder; (2) accordingly, the inhibition of hippocampal protein synthesis blocks reconsolidation of remote contextual fear memory; (3) strikingly, retrieval of remote contextual fear memory becomes hippocampus-dependent again following long-duration of memory retrieval (Ishikawa et al. 2016). These observations suggest that a long-duration reminder is sufficient to re-engage the hippocampus, even at remote time points, compared with initial memory formation.

PTSD patients show frequent and spontaneous retrieval of traumatic memories (flashbacks). Therefore, it is possible that robust traumatic memories remain hippocampus-dependent without being rendered hippocampus-independent since spontaneous and continuous retrievals may continue to induce hippocampus-dependent destabilization/reconsolidation loops (see above). It is also likely that forgetting can be induced without manipulation of traumatic memories such as long-duration memory retrieval described above.

The promotion of hippocampal neurogenesis is thought to enhance forgetting of not only traumatic memories selectively but also other hippocampus-dependent memories. This represents a disadvantage with respect to PTSD treatment since even important memories will be forgotten as a result of increased adult hippocampal neurogenesis although there may be protective mechanisms that act to maintain important memories. However, targeting forgetting may still be beneficial for PTSD treatment if patients show severe PTSD symptoms, even though forgetting of any hippocampus-dependent memories would be promoted during the treatment of PTSD. Therefore, the advantages and disadvantages of promoting forgetting processes in the PTSD treatment should be carefully considered. However, as supported by the observation that exercise enhances forgetting (Ishikawa et al. 2016), the enhancement of hippocampal neurogenesis may not artificially erase the memories but simply promote the mechanism of forgetting that animals innately possess, and MEM treatment or exercise facilitates the forgetting process faster than under normal conditions.

Our findings suggest that targeting forgetting processes can be a potentially viable alternative or adjunct to extinction and reconsolidation-based approaches. Interventions that promote the forgetting of traumatic memory may be useful for improving the efficacy of PTSD treatment.

## Conclusion

PE therapy is an effective cognitive therapy for PTSD. While interventions to treat PTSD have focused on reconsolidation/destabilization and extinction, it has been suggested that induction of forgetting could shorten and/or improve PE therapy since continuous/repeated traumatic retrieval is not required for the enhancement of forgetting of traumatic memories by neurogenesis enhancers. Thus, interventions that promote forgetting may represent an alternative approach for modifying traumatic memories. To improve PTSD therapy via the targeting of memory processes, it will be necessary to further investigate the mechanisms underlying fear memory regulation at the molecular and cellular levels, so as to identify more efficient targets that can enable the artificial regulation of memory processes. Additionally, the simplest means of identifying novel therapeutic targets for the modification of traumatic memories would be to utilize existing medications, such as MEM, that have been used for the treatment of Alzheimer’s disease. Furthermore, it is also important to develop valid animal models of PTSD as several laboratories have tried to develop PTSD models using rodents (Rau et al. 2005; Ritov et al. 2016). Fear conditioning is a useful model for traumatic memory but traumatic memory associated with PTSD is presumably more complex than the contextual fear memory. It would also be interesting to examine the effects of hippocampal neurogenesis enhancers on pathophysiological alterations linked with a PTSD-like state (i.e., depression and/or anxiety-like behaviors).
